# A smartphone ocular alignment measurement app in school screening for strabismus

**DOI:** 10.1186/s12886-021-01902-w

**Published:** 2021-03-25

**Authors:** Wenbo Cheng, Marissa H. Lynn, Shrinivas Pundlik, Cheryl Almeida, Gang Luo, Kevin Houston

**Affiliations:** 1 Department of Ophthalmology, The First Affiliated Hospital, Xinjiang Medical University, 137 Liyvshan Road. Urumqi, Xinjiang, 830000 China; 2 Schepens Eye Research Institute, Massachusetts Eye & Ear, Harvard Medical School, Boston, MA USA; 3 William G. Vinal School, Norwell, MA USA

**Keywords:** Strabismus, School screening, Smartphone application

## Abstract

**Background:**

Strabismus is the leading risk factor for amblyopia, which should be early detected for minimized visual impairment. However, traditional school screening for strabismus can be challenged due to several factors, most notably training, mobility and cost. The purpose of our study is to evaluate the feasibility of using a smartphone application in school vision screening for detection of strabismus.

**Methods:**

The beta smartphone application, EyeTurn, can measure ocular misalignment by computerized Hirschberg test. The application was used by a school nurse in a routine vision screening for 133 elementary school children. All app measurements were reviewed by an ophthalmologist to assess the rate of successful measurement and were flagged for in-person verification with prism alternating cover test (PACT) using a 2.4Δ threshold (root mean squared error of the app). A receiver operating characteristic (ROC) curve was used to determine the best sensitivity and specificity for an 8Δ threshold (recommended by AAPOS) with the PACT measurement as ground truth.

**Results:**

The nurse obtained at least one successful app measurement for 93% of children (125/133). 40 were flagged for PACT, of which 6 were confirmed to have strabismus, including 4 exotropia (10△, 10△, 14△ and 18△), 1 constant esotropia (25△) and 1 accommodative esotropia (14△). Based on the ROC curve, the optimum threshold for the app to detect strabismus was determined to be 3.0△, with the best sensitivity (83.0%), specificity (76.5%). With this threshold the app would have missed one child with accommodative esotriopia, whereas conventional screening missed 3 cases of intermittent extropia.

**Conclusions:**

Results support feasibility of use of the app by personnel without professional training in routine school screenings to improve detection of strabismus.

**Supplementary Information:**

The online version contains supplementary material available at 10.1186/s12886-021-01902-w.

## Background

Strabismus develops during childhood in an estimated 3–8% of the U.S. population and is the leading risk factor for amblyopia [1, 2]. If detected and treated early, visual impairment can be minimized; therefore early detection of strabismus in young children is important to ensure that treatment is administered as soon as possible [3, 4].

Given the importance of early detection, widespread school screenings for strabismus are critical; however, carrying out these screenings can be challenging. Traditionally, school nurses without specific training in eye care perform initial screening. School nurses are able to check for amblyopia using visual acuity and stereopsis, but they are not trained to conduct a cover test, which is the standard means of diagnosing and directly measuring strabismus. Measurement of strabismus with the cover test requires the examiner to detect eye movements as small as 1Δ (0.57°) and adjust a prism bar until the eye movements are neutralized, while simultaneously alternately covering and uncovering the eyes and monitoring the child for proper fixation. It would be challenging to train all the nurses and pediatricians to perform a cover test for strabismus detection and so screening protocols opt for the simpler stereo testing (typically Randot), which can miss cases of intermittent strabismus. Direct evaluation of strabismus (rather than relying on a stereopsis test) is particularly important in intermittent strabismus, where stereopsis can be normal or near normal causing most cases to be missed [5, 6].

In 2002, the American Academy of Pediatrics strongly endorsed the development of cost-effective image-based screening (photoscreening) as a means to extend screening to all children [7]. The red reflex (Bruckner) [8] and the corneal reflex (Hirschberg) techniques are the two most common strabismus photoscreening methods. The red reflex method compares the brightness of the “red eye” flash artifact (which modern cameras aim to eliminate), with the strabismus eye being a lighter or brighter red color. This method can detect both refractive error and strabismus, but cannot quantify the magnitude of the strabismus, e.g., Medical Technology Inc. Photoscreener (Cedar Falls, IA) and iScreen Vision Screener (Memphis, TN) [9, 10]. The photographic Hirschberg method utilizes the relative position of the primary corneal reflection generated by the flash, which is deviated in the strabismic eye and can therefore measure the magnitude of the strabismus using linear deviation converted into an angular deviation, which has been referred to as the Hirschberg Ratio [11, 12]. Some commercially available stand-alone photoscreeners utilize both of the methods, e.g. Welch Allyn Spot Vision Screener (Skaneateles Falls, NY) and Plusoptix Vision Screener (Atlanta, GA) [13, 14]. These photoscreeners have not been widely adopted by school districts, possibly due to cost. A smartphone based strabismus screening app could reduce costs while making screening at home also possible. Presently there is an app which uses the red reflex method, but it does not include the photographic Hirschberg method to give a strabismus angle measurement (Gocheck Kids, Scottsdale, AZ) [15]. We report here the evaluation of a new app called EyeTurn (Massachusetts Eye and Ear, Boston, MA) for school strabismus screening which utilizes the photographic Hirschberg method (but not the red reflex method). In a recent clinical study, the app was able to measure constant and intermittent strabismus with a high agreement with PACT method [16].

A school nurse was invited to use the app during the routine school vision screening and the app measurement results were compared with conventional prism alternating cover test (PACT) performed by two of the authors (WC and KH).

The primary objective was to evaluate the feasibility of using a smartphone application in school vision screening for detection of strabismus and to collect feedback from the nurse about her experience using the application to qualitatively analyze the user and student experience. Secondary objectives were to determine the best threshold for optimizing sensitivity and specificity of the app for detecting strabismus in school screening.

## Methods

This study used a cross-sectional design. The protocol was approved by the institutional review board of the William G. Vinal School, Norwell, MA, and conducted in accordance with the tenets of the Declaration of Helsinki. Written Informed consent was obtained from one parent of the subjects following detailed explanation of the nature and possible consequences of the study. Inclusion criteria included being a student at the school undergoing traditional school screening. This study was performed in three stages: 1) Standard school screening (acuity and stereo testing) followed by the app; 2) review of the images offsite and flagging for in-person assessment; 3) in-person evaluation.

**Fig. 1:**
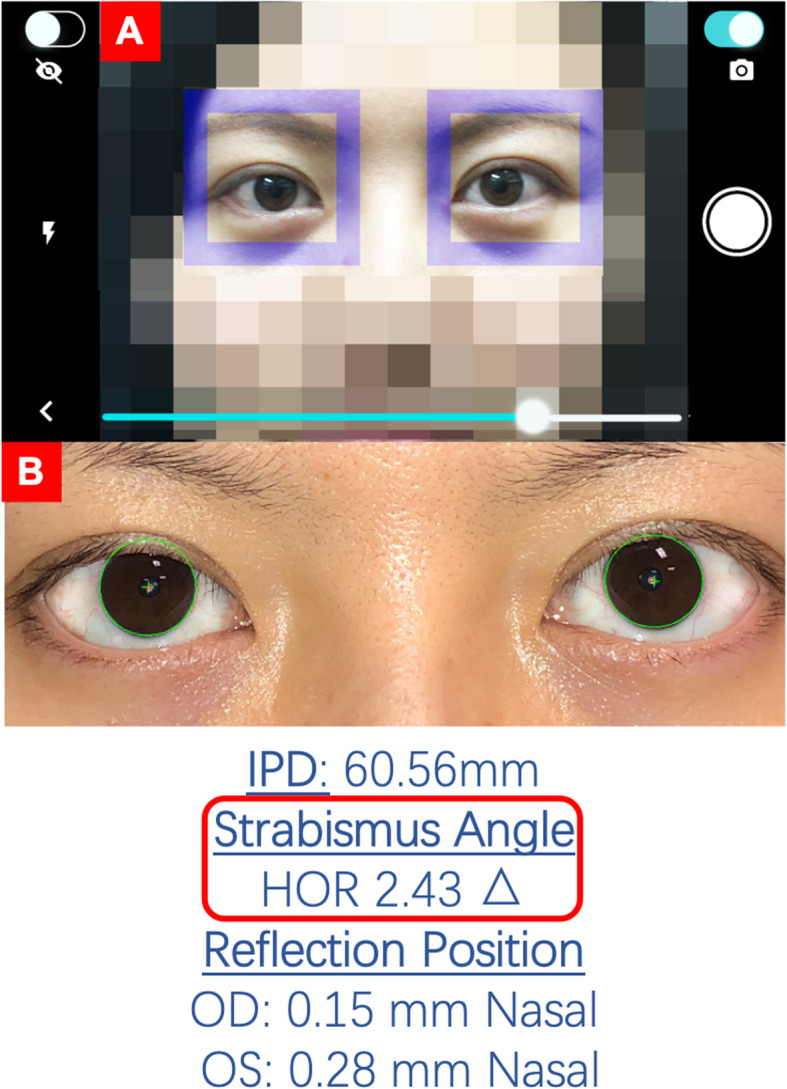
The image capturing interface. The pair of purple boxes (**a**) can be used to frame the eyes to make sure images are captured at a consistent distance and without head or eye tilt. Strabismus angle (**b**) is obtained at the time the image is captured. IPD: interpupillary distance. HOR: horizontal strabismus angle

Stage 1: A school nurse performed the standard school vision screening including visual acuity with tumbling E’s at 10 ft. and OPTEC 2000 machine for near acuity and stereopsis test. Afterwards, for those whose parents consented, the nurse made 3 consecutive measurements with the app on her own iPhone 7. To capture pictures of the children’s eyes, the nurse had students sit, stare directly at the smartphone camera flash (iPhone 7, EyeTurn Version 1.0), and then used the mobile application to align the children’s eyes within 2 boxes displayed by the app overlaid on a live image from the phone camera (Fig. 1). For each child, the nurse attempted 3 pictures. There is a video capture mode in the app, which requires the operator to perform cover/uncover while recording video, but this was not used. The nurse only used snapshot mode in measurement.

Stage 2: An ophthalmologist (WC) masked to gender, age, and any prior known ocular history of the children reviewed the images offsite which were de-identified by the app automatically by cropping the image to save only the eye region (Fig. 1b). Data was provided confidentially by the school’s nurse through secure email. Based on a predefined protocol (see supplement), the ophthalmologist determined whether the software correctly located image features such as iris and corneal reflection. Measurements with incorrect feature detection were rejected for analysis but documented for reporting. The school was not willing to allow the examiners to test every child in-person for logistical reasons and so a criterion for in-person testing was set at 2.4Δ, as measured by the app [16]. This criterion was selected based on the root mean squared error (RMSE) of the app in a prior evaluation study [16], and is well below the AAPOS suggested referral criteria (8Δ) [17]. If the app measurement error is due to random noise, positive cases are unlikely in students with app measurements smaller than RMSE, at the level of 1% probability of a false negative for patients who meet AAPOS 8Δ strabismus criterion (the probability will be even lower for larger strabismus). If the app error is due to other constant factors, for instance, small angle strabismus manifestation during app measurement, the app may miss some positive cases. As the in-person re-test threshold was so low (2.4Δ), we expected that any children whom tested positive by the conventional screening that may have been missed by the app were also included in the in-person evaluation.

Stage 3: Identified students were evaluated in-person with the unilateral and PACT performed at near by the pediatric ophthalmologist (WC) and optometrist (KH). As the version of the app used in the study could not perform a distance measurement, cover testing was only performed at near. The unilateral CT confirmed whether or not there was a tropia but was not prism neutralized (the PACT was used to quantify the deviation (Table 1)). All cases classified as ground-truth positive manifested a tropia on unilateral cover, at least intermittently, and had a PACT >8Δ. Extra-ocular motility and near point of convergence testing were also performed [18]. Threshold for positive cover test screening was set at >8Δ (at least intermittently), as recommended by the American Association for Pediatric Ophthalmology and Strabismus (AAPOS) [17]. The examiners were masked to the app values during the cover testing; however, they did know that the child met the criterion level with the app and so were looking closely for strabismus.

**Table 1:** Strabismus Subject Characteristics

Subject	gender	Ethnicity	PACT (△)	EyeTurn (△)	NPC (cm)	Diagnosis
1	male	A/C	10 IXT	3.03	20	IXT, CI
2	male	A/C	14 IXT	4.07	20	IXT, CI
3	male	A/C	10 IXT	3.18	2	IXT
4	female	Caucasian	18 XT	8.97	Constant XT	Congenital 4th nerve palsy with constant XT
5	female	Caucasian	25 ET	20.19	10	Constant ET
6^a^	female	Caucasian	14 ET	2.52	6	IET

^a^Esotropia case was caught by PACT but missed by the app with 3.0△ as the optimum threshold (evaluated by ROC curve). Of the 6 subjects, 5 were also diagnosed as convergence insufficiency (CI) by near point of convergence (NPC)

*A/C* Asian/ Caucasian, *XT* exotropia, *ET* esotropia.

Strabismus criterion: Manifest strabismus > 8△(recommended by AAPOS) [17].

CI criterion NPC ≥ 6 cm [19].

### Subjects

The study was conducted between November 2017 and January 2018 at an Elementary School in Norwell, Massachusetts, US, with a population of 10,506 at the 2010 Census. All parents at the school in grades 1 to 5 (520 students) were offered participation for their child by the school sending a study information sheet and consent form in the mail.

The school nurse provided de-identified app data and cropped eye images for 133 students, grades 5 to 1. Age, gender, and race were not recorded in the app data therefore researchers were masked to these characteristics during review, later obtained from the nurse for the positive cases as reported in Table 1. Iris color was documented from review of the eye-cropped images finding 55 (41.4%) dark colored irises (brown or hazel) and 78 (58.6%) light colored irises (green or blue), and analyzed for any differences in measurement success with the hypothesis that dark iris border may be more easily and reliably detected by the software.

### Measurement of eye alignment using the app

When eye images are taken, a pair of purple boxes appears on the interface to help guide positioning and minimize head tilt. The strabismus angle is obtained at the time the image is captured (Fig. 1).

**Fig. 2:**
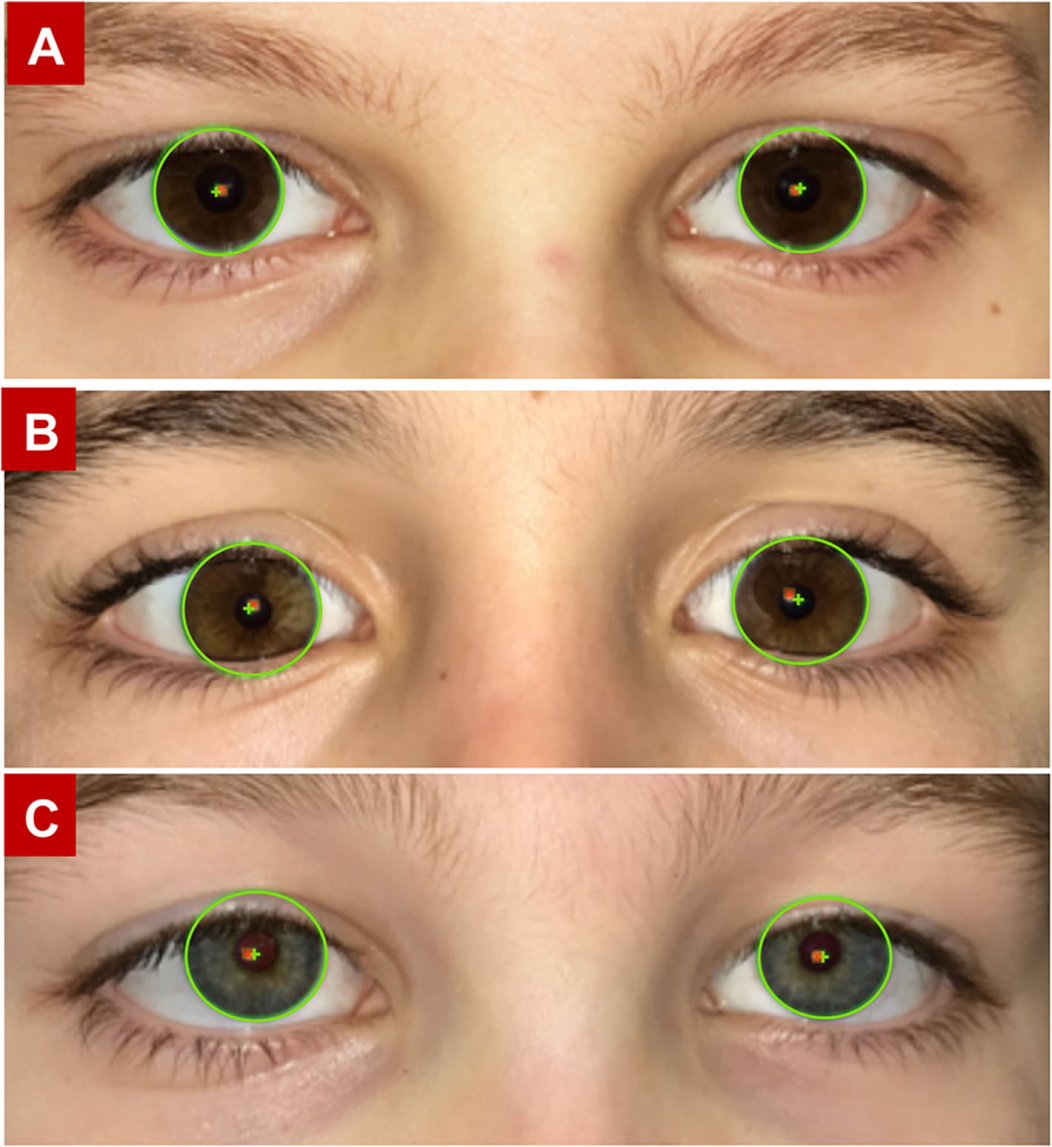
The green circle marks the border of the iris and the green cross shows the center. The red dot is the corneal reflection. In **a**, the distances between limbus center and corneal reflection (DLR) of each eye are equal and small (0.38 mm for OD and 0.49 mm for OS). Both corneal reflections are located nasal to the limbus centers with no obvious strabismus measured. In **b** and **c** there are substantial differences in DLR. In **b**, 0.28 mm OD and 0.83 mm OS, with a strabismus angle calculated at 10.51△ (exodeviation). In C the corneal reflection is located in different sides of the iris center and the strabismus angle is calculated at 20.19△ (esodeviation). **b** shows a suspected exotropia while (**c**) is a suspected esotropia

A school nurse with 20 years of experience conducting traditional school screenings took all the measurements with the application. Some participating students were already known by the school nurse to have vision problems. Prior to the study the school nurse received a brief in-person tutorial and one trouble-shooting session after the first 3 classes were screened. The nurse placed the iPhone at a typical reading distance approximately 40 cm in front of the subjects’ eyes for the measurements. All measurements were taken in the nurse’s office with overhead fluorescent lights turned on using her own personal iPhone 7. The subjects were asked to remove their glasses (of the 133 subjects, 17 subjects needed glasses to correct refractive error) and look at iPhone flash. A successful measurement was defined by the ability of the application to detect the border of the limbus and the corneal reflections (Fig. 2).

### Unilateral and prism alternate cover test (PACT)

Two eye doctors with 6 and 17 years of clinical experience in pediatric ophthalmology or optometry performed the cover testing. Subjects were asked to focus on a 20/30 size row of letters 30 to 40 cm away. The cover testing was performed as is done in standard practice [20]. For unilateral cover testing one eye was covered while watching the uncovered eye for a refixation movement, uncovered, and then repeated for the other eye. Next the PACT was performed. Prisms of increasing strength (prism bars, Bernell, Mishawaka I.N.) were placed in front of each eye with the base opposite the direction of deviation. As stronger prisms were added, the amplitude of ocular re-fixation movement gradually decreased. When ocular re-fixation movements were extinguished, the angle of deviation was taken as the strength of the prism.

### Data analysis

Analysis was conducted using SPSS (version 23.0; IBM Corp., Chicago, IL.) and STATA (version 14.2; STATA Corp., College Station, Tx). To determine if the app iris and corneal reflection fitting success rate was better for dark irises than light, random effects multiple logistic regression analyses were performed, one each for failure due to eye closing, eye detection failures, iris detection misfitting, and corneal reflection misfitting with iris color (light or dark) and order of the school Class tested as covariates. Significance for Class would suggest there was a learning effect of the nurse or an effect of age (nurse started with 5th grade class and tested in order with 1st grade tested last). The optimum threshold for sensitivity and specificity for the app was determined with the prism cover test as the ground truth using ROC analysis. Positive and negative predictive values were also calculated. Pearson’s correlation coefficients (r) were used to analyze the linear fit of the relationship between variables. The level of statistical significance was set at *P* < 0.05 for all analyses.

## Results

A total of 377 images from the 133 subjects were available for analysis, of which 290 (76.9%) had a usable measurement with proper iris and corneal reflection detection in both eyes. 125 (93.3%) subjects had at least 1 successful measurement; 90 (66.2%) had 2; 48 (35.3%) had 3, and 9 (6.7%) had no successful images captured.

The most common issue reported by the nurse was sensitivity to the flash resulting in eye closing, and image analysis errors resulting in wrong identification of the iris border, corneal reflection or both. At the troubleshooting session (after 3 classes were screened), the main issue addressed was that the images were off center due to centering of the phone to the student’s face rather than the camera, which can cause the image size to vary across the horizontal axis and may induce measurement error.

Overall the nurse reported her experience using the application as positive and intuitive. Contrary to the nurse’s report, eye closing was documented in images as the cause for measurement failure only 13 times representing 3.5% of all images (13/377), occurring in 1.9% with dark irises (3/154) and 4.5% with light irises (10/223) (Table 2), which was not a significant difference, *p* = 0.21, z = 1.27. There were 48 iris border misfittings representing 12.7% of all images reviewed (48/377), 12.3% of which were dark irises (19/154) and 13% of which were light irises (29/223), also not a significant difference *p* = 0.80, z = 0.25. Reflection misfitting was found to occur when the iris was detected accurately but the reflection was misidentified, typically as a secondary reflection of the flash at the tear meniscus of the inferior lid margin or off the top the eye lid skin presumably caused by oily skin. This occurred in 8/154 dark iris students (5.2%) and 13/224 light iris students (5.8%), which was not a significant difference, *p* = 0.77, z = 0.30. Failure of the software to detect the eye, the first step in processing, occurred in 4 dark and 1 light irises (2.6 and 0.5% respectively), also not significant *p* = 0.11, z = − 1.58. Eye closing, eye detection, iris misfitting, and corneal reflection misfitting represented 14.9, 5.7, 55.2, and 24.0% of measurement failures (*n* = 87), respectively. Date of data collection was not significant (all *p* > 0.27) suggesting measurement success did not require much practice.

**Table 2:** Inventory of images reviewed and app software failures

	Eye Closing	Eye Detection Failure	Iris Misfit	Reflection Misfit	Total Unsuccessful	Successful
Dark Irises	3 (1.9%)	4 (2.6%)	19 (12.3%)	8 (5.2%)	34 (22%)	154 (40.8%)
Light Irises	10 (4.5%)	1 (0.5%)	29 (13%)	13 (5.8%)	53 (23.8%)	223 (59.2%)
Total	13 (3.5%)	5 (1.3%)	48 (12.7%)	21 (5.6%)	87 (23.1%)	377

None of the differences between light and dark irises were statistically significant.

Based on the app, the median strabismus angle for the 125 subjects who were successfully measured were 2.89△ for XT (95% reference range: 2.5 ~ 3.78), and 2.25△ for ET (95% reference range: 1.96 ~ 4.71). The largest strabismus angles were 28.54 for XT, and 20.19 for ET (Fig. 4). Using the screening threshold 2.4△, 40 subjects were flagged for in-person testing. Among them, 6 subjects were confirmed to have strabismus, including 3 intermittent exotropia and 1 constant exotropia (mean 13△, range 10△ to 18△, *n* = 4), 1 constant esotropia (25△) and 1 intermittent accommodative esotropia (14△) (Table 1). The other 34 in the cross-sectional in-person testing sample did not have strabismus on unilateral cover test but according to PACT, 5 had esophoria (mean 4.40△, range 2△ to 6△), 1 had orthophoria, and 28 had exophoria (mean 4.11△, range 2△ to 8△).

**Fig. 3:**
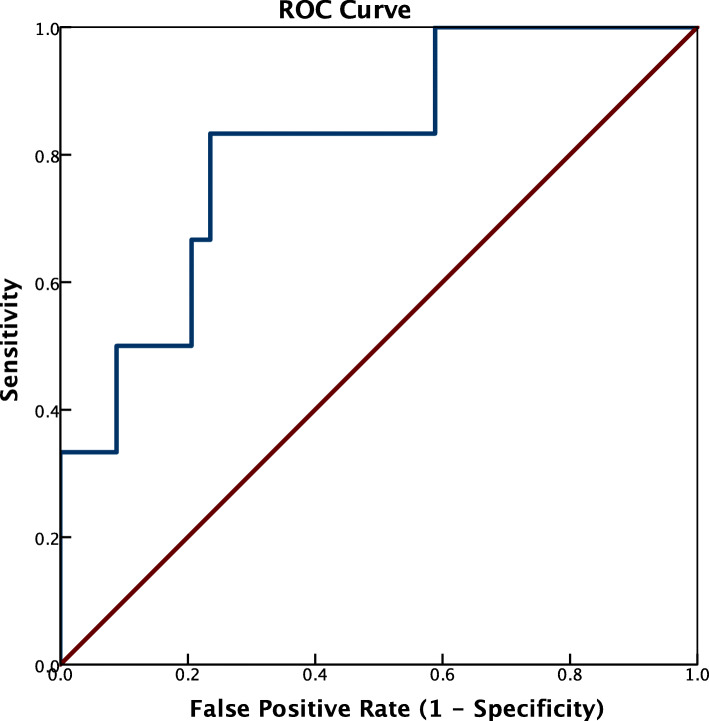
ROC curve, i.e. sensitivity vs. false positive rate, as it varies by prism diopter threshold (solid blue staircase shaped line). The optimum threshold used to indicate a positive screen (closest point to the top left corner) was determined to be 3.0△. Sensitivity and Specificity with this threshold were 83.0 and 76.5% (1–0.235) respectively, marked by the gray dashed line

Based on the ROC curve (Fig. 3), the optimum threshold for the app to detect strabismus was determined to be 3.0Δ with the best sensitivity (83.0%) and specificity (76.5%). With this criterion, there were 5 true positives, 8 false positives and 1 false negative (subject with 14△ accommodative esotropia), as shown in Table 3. The 5 true positives included 4 cases of exotropia and 1 case of constant esotropia. Of the 8 false positives, 2 had convergence insufficiency with a receded NPC.

**Table 3:** Sensitivity, Specificity, and positive and negative predictive value of the app when using the 3.0Δ threshold

	PACT Positive	PACT Negative	Total	Predictive Value
App Positive	5	8 (false)	13	PPV = 38.5%
App Negative	1 (false)	26	27	NPV = 3.7%
Total	6	34		
	Sensitivity = 83.3%	Specificity = 76.5%		

Using the 3Δ threshold, one subject with strabismus (accommodative esotropia) would have been missed by the app (Table 1); and 5 subjects with strabismus would have been accurately identified with the application. On the other hand, based on the standard school screening protocol, 5 cases of strabismus were missed with only one subject detected as abnormal (constant exotropia with 18△), which was also detected by the app. It is likely the school screening missed the esotropia cases because the children had been identified and treated previously, and were wearing their glasses during the conventional vision screening tests. Therefore, the conventional screening really only missed the 3 IXT cases.

**Fig. 4:**
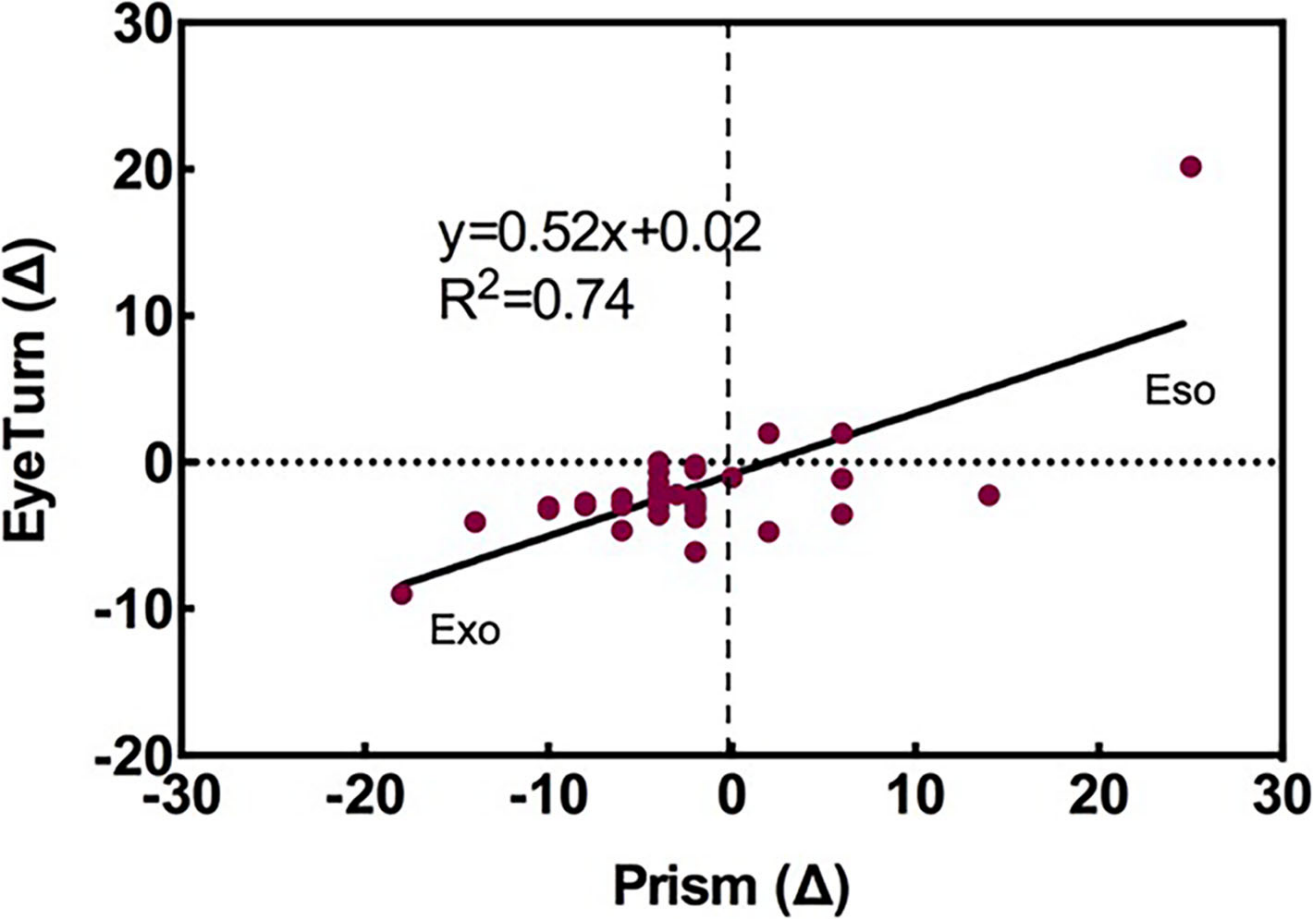
A positive correlation was found between the deviation degree measured by the app and prism alternating cover test (*R*^2^ = 0.74, *P* < 0.001). The horizontal axis represents the deviation in prism diopters measured by PACT (prism alternating cover test). The vertical axis represents the deviation in prism diopters measured by the app. Positive values are esodeviation and negative values are exodeviation. There are a few data points which overlap

A positive correlation was found between the deviation degree measured by the app and prism cover test (R^2^ = 0.74, *P* < 0.001) (Fig. 4).

## Discussion

This was a study to evaluate the feasibility and value of a adding a novel strabismus screening smartphone application to conventional school vision screening, as well as calculating threshold value for a positive screening to maximize sensitivity and specificity.

The findings suggest that a school nurse with no special training in the measurement of strabismus can easily add the EyeTurn app to their iPhone (available for iphone 6 to 8 or an iPad pro owned by the school), use the app alongside conventional school screening, and improve the identification strabismic children ages 6–12 educated in elementary school. There was value in adding the app to the screening with 3 children identified who were missed by conventional screening and had a previously undiagnosed intermittent exotropia. They were subsequently referred by the school nurse to an eye doctor for management of the intermittent strabismus.

### Discussion of functionalities, settings, and methods in lieu of the results

The app will presumably detect the strabismus without the glasses and the recommendation for referral can be ignored by parents when the issue has already been identified. The nurse was instructed to use the app at 30 cm to 40 cm, which is ecologically valid for school work and learning which primarily involve these near distances. Distance does not need to be precisely controlled and there is no need to add any landmark / indicating marker of known size in the plane of corneal reflection (e.g. credit card stripe as is used in some pupillary distance apps) as the app auto-calibrates for distance [16]. There may be concern that the Hirschberg ratio (HR) population variability is too great to use the computerized Hirschberg method without individual measurement. Measuring the HR with the iPhone has been done on a smartphone via a monocular measurement at various gaze positions of known eccentricity in each eye [16], and requires precise fixation but is otherwise not difficult to obtain; however, a prior study with the app found that a population norm HR was of essentially equivalent accuracy [16]; therefore, this extra step which could jeopardize feasibility is not needed. The version of the EyePhone app used in this study was limited to measurement of strabismus in horizontal direction only, but our data here suggest it is probably not needed for screening in elementary school children. Future studies will involve measurement of eye deviation in both horizontal and vertical direction.

### Discussion of image capture and measurement success and failures

The results of the study suggest that it is not difficult to obtain at least one successful image for over 90% of elementary school students tested, given 3 attempts. The most common issue reported by the nurse was sensitivity to the flash resulting in eye closure and the failure of the software to automatically and accurately locate the eyes and limbus; however, of the 87 images with fitting failures, only 13 were due to eye closing. Therefore while the flash may have been bothersome to some children it did not seem to have a substantial impact on successful measurement. Iris misfitting was a greater issue representing a little more than half of all measurement failures. The app did equally well at measuring strabismus in children with light irises as in dark irises, which is encouraging. The overall image capture failure rate of 23% can be compared to a prior study of the GoCheckKids app at about 12% [15]. This seeming difference is somewhat surprising since both methods require flash carrying the risk of eye closure, and automatic processing in both apps requires iris border detection. It is possible that precise iris fitting is less critical for the GoCheckKids’s red reflex method.

### Discussion of the value of adding automated Hirschberg to amblyopia screening

Combination of the automated Bruckner and Hirschberg methods may be possible in an app as is done for multiple dedicated photoscreeners (see introduction), although this is likely to increase image processing time potentially creating a feasibility issue in school screening. Adding strabismus detection with photographic Hirschberg may not be of value if the only issue of concern is amblyopia detection, which has thus far been the principal target of AAPOS and AAP. However, stakeholder interest (educators, OTs, parents) in the negative impact of intermittent exotropia and convergence insufficiency on academic performance is growing and may drive new policies around targeted screening for these conditions if a feasible method exists [21].

### Discussion of the 1 case of strabismus missed

The app was successful in detecting four cases of intermittent exotropia and one case of constant esotropia. When the optimum threshold was determined (ROC analysis) and reset to 3.0△, the EyeTurn app would have narrowly missed a case of 14Δ accommodative esotropia (as determined by in-person assessment with 20/30 letter as fixation). This was the only case of strabismus that the app would have missed in this cross-sectional sample. The smaller amount of esotropia with the app compared to ground-truth may be because using the camera lens as the fixation target did not introduce much accommodative demand. Similar occurrences were reported in a study about long-term follow-up in accommodative esotropia [22]. Providing a 20/30 letter target on the phone near the camera lens may solve this problem. Conventional school screening only identified one case of strabismus (exotropia) with decreased stereoacuity, which was also identified by the app. This suggests that traditional school screening may miss many cases of intermittent strabismus where visual acuity and stereoacuity are good [23].

The sensitivity and specificity of (76.5 and 83.0%) measured in this study can be compared to a study with the Welch Allyn Spot Vision Screener’s ability to detect strabismus (50 and 96%) where a trained non-medical staff or volunteer took the images with the ground truth coming from those children whom ultimately followed up with an eye doctor [24]. In another study of the Spot screener in detecting strabismus, this time in a pediatric ophthalmology office, sensitivity of (77 and 93%) were reported [25], but it should be noted that intermittent cases were not considered as positive in that study, which likely inflates the values. A prior study of the GoCheckKids app was similar to this study only in that it was also deployed on a phone, but used the automated Bruckner method, and was conducted in a different age group (ages 6 months-6 years) finding the sensitivity of (74.6%) and specificity of (67.2%) for comparison [26]. The Plusoptix photoscreener is also used for detecting strabismus in children (2–14 years) with the sensitivity (40.7%) and specificity (98.3%) respectively [27].

### Discussion of the app for dissociated measurements

Sensitivity to bright light is a characteristic often associated with intermittent exotropia due to a “dazzling” of the retina and disruption of fusion [28, 29]. This kind of disruption after dazzling can last more than 0.8 s, which is long enough for the app (about 0.2 s) to capture the eye misalignment [30]. In this study, Therefore the flash may be helpful in dissociating the eyes of children with intermittent exotropia and convergence insufficiency, revealing the conditions as our data suggests. However, the strabismus degree got may be smaller than the real value because of without cover/uncover test, which could be improved by the update design of the app. Of the 8 false positives, 2 of those were found to have convergence insufficiency by in-person testing which may be a detriment to near work and learning [31], other 6 may be big kappa angle causing overestimated. Our findings suggest screening for convergence insufficiency may be possible with this app, and could be valuable given its negative impacts on near work activities important for learning which are treatable with vision therapy [32].

## Limitations

The true false negative rate cannot be known in this study, because the school could not, for logistical reasons, allow in-person measurements on all the children who were screened with the app. Therefore, there may be a couple children that were missed by both the app and the traditional screening. In addition, this was not a random sample so it is unknown if it was representative of the entire school. In terms of eye detection errors, which can be due to eye closing or other sources of interference, need more investigations to solve.

## Conclusions

This study demonstrates that it is feasible for non-eye care professionals such as a school nurse to perform strabismus school screening using a smartphone application, and that the app improved the ability of the screening to catch cases of intermittent exotropia (and possibly convergence insufficiency), being better than conventional school screening alone. While the specificity (76.5%) and sensitivity (83%) are promising, further studies are needed to confirm the findings. Technical improvements on the application are underway to address the iris and corneal reflection detection failures.

## Supplementary Information

**Additional file 1.**

## Data Availability

Data supporting the results reported in the article are not public but can be accessed after communicating with the corresponding author.

## Abbreviations

PACT: Prism alternating cover test
ROC curve: Receiver operating characteristic curve
RMSE: Root mean squared error
