# Supplementary material for: A smartphone ocular alignment measurement app in school screening for strabismus
Source: BMC Ophthalmol. 2021 Mar 25;21:150. doi: 10.1186/s12886-021-01902-w (PMC7992982; doi:10.1186/s12886-021-01902-w)
Supplement: Supplementary file 1 — Additional file 1. [file 12886_2021_1902_MOESM1_ESM.docx]

EyeTurn School Screening Data Processing Protocol

1. Open the most current version of the data spreadsheet from the school nurse’s phone. The spreadsheet comes from EYEnexo online data base. This file is continuously updated (information from people using EyeTurn all over the world goes into this database automatically, so long as they have wifi connection).

1. Go to firebase.google.com or google “google firebase”
2. Click on head icon and

| 1. 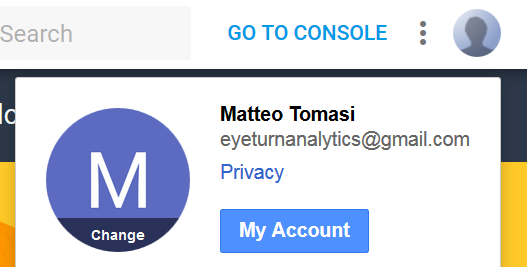 |
| --- |

1. The first time you will need to click “add account”
2. Sign in with address [eyeturnanalytics@gmail.com](mailto:eyeturnanalytics@gmail.com) and password eyeturnapplication
3. Click “go to console”
4. Click “EyeTurn”
5. Click “database”

| 1. 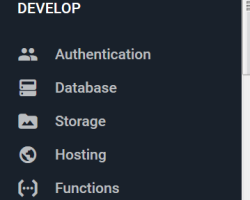 |
| --- |

1. Select dropdown “users” and find the phone named iPhone9, 1_150643060033734 and double click

| 1. 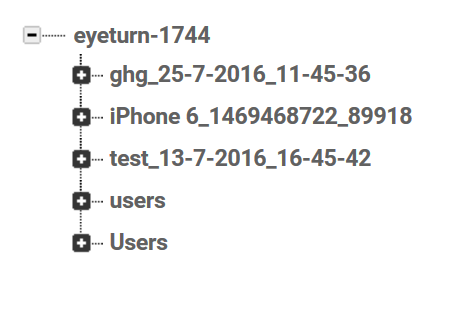 | 1. 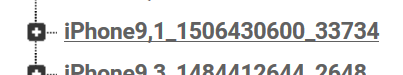 |
| --- | --- |

1. Save file to your computer by clicking the 3-dot icon and select “export JSON”

| 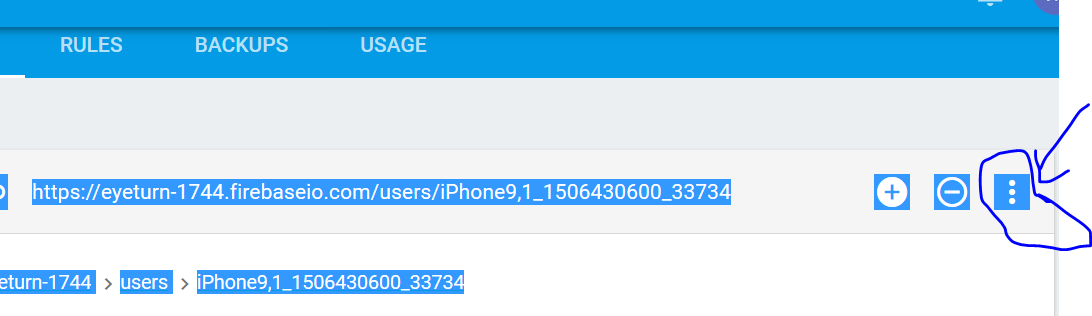 |
| --- |

1. To open the file as an excel, you will need to convert it to a .csv file
2. Search “convert JSON to csv”. The converter I used was <https://json-csv.com/>
3. Click upload and find the JSON file you just saved. Follow the prompts and you should end up with a file you can open in excel.
4. You should check this excel sheet weekly to be sure there was no additional data added
5. Open a PDF report
   1. PDF reports were sent to me from the school nurse by email. I uploaded them to where you see them now.
   2. Go to the pelishare server -> projects -> strabismus app -> school screening
      1. The path on my computer was this: P:\Projects\Strabismus App\SchoolScreening
   3. Each PDF file is one class of about 25 children.
6. Copy PDF files to your local computer
7. Open the earliest screening data (password is “Vinal”, which is part of the file name. report_Other_2017_9-11-2017_9-11-47
   1. The data is in day-month-year, so the above screening date was 5 November 2017
8. Now that the files are open, the first thing we need to do is make sure the data automatically uploaded to the EYEnexo database. We expect that there will be some files partially or completely missing. It is also possible the nurse forgot to send me something. In that case there may be a record of exam in the EYEnexo spreadsheet but no corresponding PDF report.
   1. Column A is the PDF name, file B should match the time stamp on each individual image in the PDF.

| 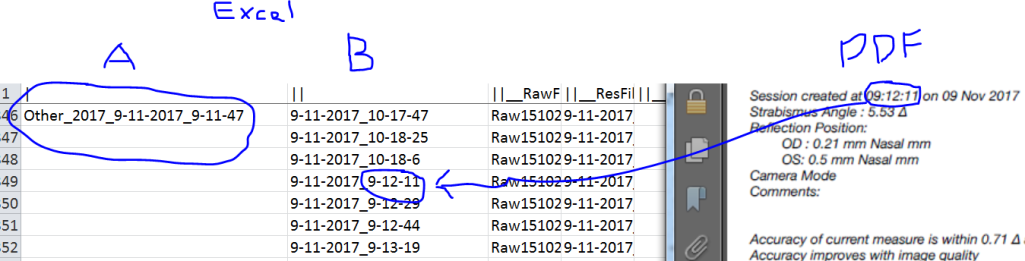 |
| --- |

- 1. Add a column titled verified and another titled positive screen. If the screening value is positive (greater than 2.4Δ) enter a “1”, otherwise enter a “0”. If there was some problem (e.g. iris did not fit properly) enter a zero.

| 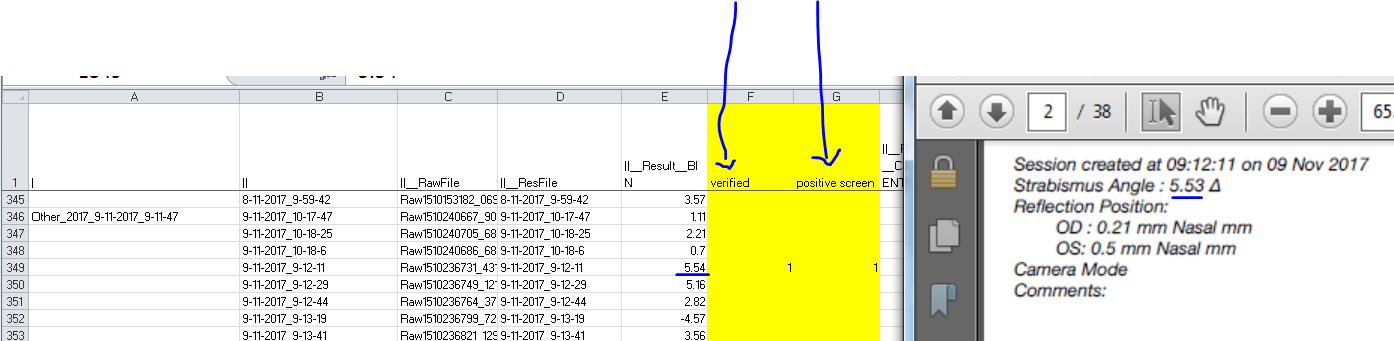 |
| --- |

- 1. If you don’t find an entry matching the image in the PDF, insert a new row and enter as much info as is in the PDF and whether or not it was a positive screen.

1. Make a screen shot of the image and put into a folder titled “positive screen”. Name the image according to the time stamp.
2. In a word document, keep a running list of positive screens by patient. The nurse will need the date, time, and order to identify the child. By order, I mean 3^rd^, 4^th^, 5^th^ child in sequence on that day. Each child should have 3 images. If all 3 are positive, then obviously the screening is positive and they should be seen by me. If only 1 image out of 3 is positive, you may need to make a judgment call if you think we should recommend they be seen. If so add them to the list. Put the screen shots of their exams in the list to make easy reference. It also allows me to check when I go in to exam them that the picture matches the child.

Example

1. 9 nov 2017 at 9:12am, first child tested on that day.

| 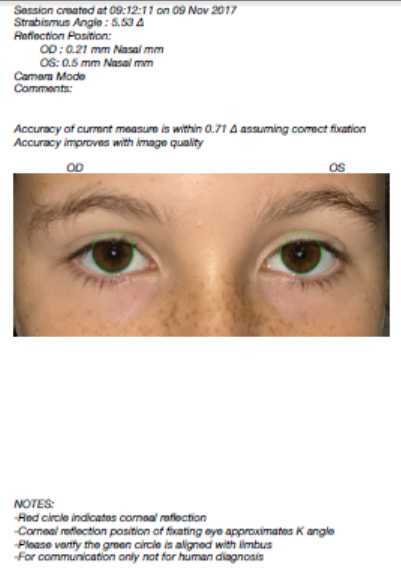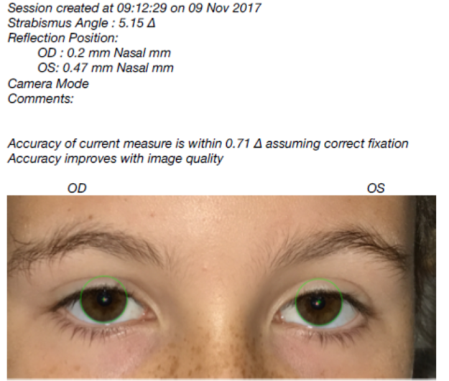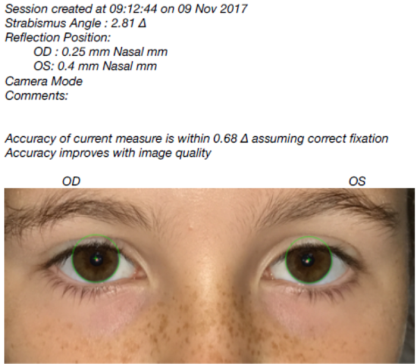 |
| --- |
